# The Effect of Animal Welfare Training on the Knowledge and Attitudes of Abattoir Stakeholders in China

**DOI:** 10.3390/ani9110989

**Published:** 2019-11-18

**Authors:** Kris Descovich, Xiaofei Li, Michelle Sinclair, Yan Wang, Clive Julian Christie Phillips

**Affiliations:** 1 Centre for Animal Welfare and Ethics, School of Veterinary Science, Building 8134, Gatton Campus, University of Queensland, Gatton, QLD 4343, Australia; xiaofei.li1@uq.net.au (X.L.); m.sinclair6@uq.edu.au (M.S.); c.phillips@uq.edu.au (C.J.C.P.); 2 College of Animal Science, South China Agricultural University, Guangzhou 510642, China; ywang@scau.edu.cn

**Keywords:** welfare, slaughter, transportation, China, attitudes, training

## Abstract

**Simple Summary:**

China is one of the world’s largest producers and exporters of meat, but the welfare of livestock is only now emerging as an important issue. This study looked at whether a “train the trainer” program could be useful in improving the knowledge of employees within Chinese abattoirs about animal welfare during slaughter and transport. Trainers were either taught as a group in a classroom or mailed the training material. They subsequently held training sessions within their own workplace and the participants were surveyed before or after training. The post-training participants were more knowledgeable about animal welfare and were more confident that they could improve welfare for the animals in their care only when they were initially taught in the classroom setting. Participants without a high school education had lower knowledge scores than other participants, and women had more positive attitudes towards animals than men. These results suggest that “train the trainer” initiatives can be successful, but that consideration needs to be given to the mode of training, the level of participant education, and the content in order to ensure that all participants gain equally from the training.

**Abstract:**

Globally, China is one of the largest producers and exporters of meat, and animal welfare is an emerging focal issue for Chinese society and for primary producers. We assessed the effectiveness of a “train the trainer” program to increase awareness of animal welfare issues in stakeholders in the livestock industries of China. Chinese abattoir employees were trained in slaughter and transportation standards in either a classroom setting or using posted materials. They subsequently held training sessions within their own workplace and the participants were surveyed either before (*n* = 161) or after (*n* = 147) their training. The post-training group had more confidence to improve the welfare of animals in their care than the pre-training group (*p* = 0.03), and also scored better on the knowledge section of the survey (*p* = 0.006) only when the facilitator was trained in the classroom setting. The participants’ knowledge of animal welfare was also affected by living area (*p* = 0.005) and education (*p* = 0.005). Participants with the least formal education (to middle school only) scored lower than all other participants. Female respondents reported more positive attitudes towards animal welfare than males (*p* = 0.009). These results indicate that training can effectively improve stakeholder knowledge on animal welfare during slaughter and transport, however, the mode of delivery has an important influence on learning success, and participant demographics, such as gender and education level, need to be considered when preparing training material.

## 1. Introduction

The social license that is implied by animal use is increasingly dependent on the provision of an adequate standard of welfare for the animals [1]. Animal welfare is an important societal concern and a component of socio-cultural sustainability, one of the four “pillars of sustainability”, along with economic, human health, and environmental sustainability [2,3]. These pillars are inter-connected, and high standards of animal welfare also protect human health, restrict disease transmission, and control financial risk through corporate social responsibility [4,5,6,7,8,9].

Over the last 50 years, much progress has been made in understanding the welfare of livestock in regions such as Europe and North America [2,10]. Animal welfare is now emerging as a focal issue in economically developing countries, particularly those which produce, consume, and export large amounts of the world’s meat, such as China and Brazil [10,11]. Much of this meat is produced using intensive farming, housing and management, which may have welfare trade-offs [10,12]. Legislation to protect animal welfare and provide minimum standards of care varies widely across the world. In many countries, regulation is minimal, comprised of voluntary standards, or is even absent, irrespective of the scale of production in that country [11,12]. For example, China is the world’s largest producer of pigs and chickens [13], however, it has no enacted legislation for the protection of livestock, despite drafting a law to this effect in 2009 [14,15]. Any progression towards high-welfare animal production could only currently be driven by a range of mechanisms other than legislation, including societal pressure, general economic development, scientific advances and innovation, retailer pressure, advocacy, and voluntary industry uptake [10,11,12,16].

Animal welfare, as a science, focuses on the experience of the animal [17], however the development and implementation of welfare standards is a human construct, given that humans are responsible for almost all decisions that affect the welfare of the animals in their care. This human element is complex, influenced by a diverse range of factors including gender, religion, nationality, personality, and affluence [9,18,19,20,21]. The interpretation of the multi-dimensional concepts that comprise animal welfare [17] is also influenced by stakeholder group membership. For example, farmers generally hold different views on the use of painful procedures (e.g., dehorning and castration) and pain mitigation in livestock management than veterinarians or the general public [22,23,24,25]. Attitudes and beliefs towards animal welfare, use, and management are further influenced by the values, experiences, norms, and interests of each stakeholder [26,27], and people may simultaneously hold multiple, competing views. For example, they may express positivity towards modern farming systems for providing low cost food or high food safety, but may have concerns about the impact of the same systems on animal welfare or local customs [28]. To forge improvements in animal welfare that are acceptable to society, the development of solutions should include all stakeholder groups, and should allow actual and perceived representation of diverse views, as well as transparency in the consultative process [2,10,29]. Humans do not always act in accordance with their own ethics or beliefs [30,31,32,33], however, attitudes feed into intentions, which typically correlate with future behavior (the “Theory of Planned Behavior”) [34]. For example, one study found that people who have more positive attitudes towards animal welfare also report eating less meat and purchasing more high-welfare products [35]. Welfare-positive attitudes amongst primary producers have also been demonstrated to have a positive trickle-down effect on animal welfare outcomes (e.g., [36,37,38]).

Information provisioning is often the modus operandi for creating societal change [39], and is an important avenue for improvement of animal welfare if there is an underlying information deficit. Unfortunately, education and training do not always result in attitudinal shifts or even knowledge uptake. For example, a 2011 study found that veterinary students had more positive attitudes towards animals after completing a degree-level course in animal behavior, welfare, and ethics, but animal science students did not [40]. Training effects on knowledge can also be affected by the existing welfare alignment of participants, as well as the species or context, and usually deteriorates over time [41]. Furthermore, in a range of fields, participant attitudes have been shown to attenuate student success in training and education [41,42,43]. Therefore, education is an important component for improving animal welfare but it is not the sole mechanism, since other factors, such as improvement of facilities and animal genetics, may be necessary to achieve good animal welfare. Training outcomes should be assessed to establish whether initiatives have the desired effect. The gauging of attitudes and knowledge is therefore an important step for creating change around animal welfare [26] and this is generally achieved using social science tools such as interviews or questionnaires [44,45,46].

China is a major producer of livestock products [47]. It is the largest producer of chickens, pigs, and sheep [13] and the world’s third largest producer of beef, although domestic consumption exceeds production [48]. Within China there has been some positive shift in attitudes towards animals, which is influenced by changes in economic position, several food safety scares, and a changing relationship with animals in general [47,49,50]. Agriculture is moving from small-scale family farms to large industrial farms, and there is a growing presence of animal protection organisations and advocacy activity [47,49]. Despite these changes, understanding of animal welfare science is still largely in its infancy in China, and to move ahead in a meaningful way, it is important to consider regional contexts and challenges, as well as knowledge, attitudes, and customs [51]. Welfare assessments have largely been developed in “Western” countries and measures may not capture challenges or opportunities outside of the regions, or breeds, for which they were developed [51]. Due to the very large number of animals produced in China, even small consistent improvements will have a large impact on welfare [51].

A small number of studies have investigated attitudes towards, and knowledge of, animal welfare in China. One 2014 study [52] surveyed the general public within Chinese communities, focusing specifically on farm animal welfare and predominantly on domestic fowl and pigs. They found that only one third of respondents were familiar with the concept of “animal welfare”. Despite this, more than 80% thought legislation for animal protection was necessary and 65% approved of mandatory laws for compelling producers “to provide better living conditions for farm animals….to help them grow and survive” [52]. Food safety appears to be an important factor in Chinese attitudes towards livestock production and may drive support for industrialized production systems [53]. However, food safety in China may also be linked to animal welfare in people’s attitudes. In one survey of the general public in China, industrialized farming was considered cruel by 20% of respondents, and many negative comments given by respondents in relation to industrial farming also related to food safety (e.g., the “overuse” of additives/antibiotics) or product quality (e.g., “bad taste”).

A third study surveyed stakeholders within the livestock industry in four Asian countries (China, Malaysia, Vietnam, and Thailand) with regards to animal transportation and slaughter [54]. Chinese participants tended to have strong intentions of making animal welfare improvements in their workplace and a correspondingly high level of confidence in their ability to do so. Importantly however, participants from all countries considered that knowledge critically underpinned their ability to create change [54]. The number of animal welfare scientists and experts working in China is growing, but is still relatively low [49], which makes the provision of targeted training difficult to accomplish without international collaboration. Over the last 5–10 years, this has been increasing through avenues such as the international Animal Welfare Standards Project (AWSP) (www.animalwelfarestandards.org) but given the vast number of stakeholders in this industry within China, the feasibility of delivery methods for vocational training should be considered. This is not a challenge restricted to animal welfare. Distance learning is now offered by most universities, and while many studies suggest remote delivery of qualifications result in positive learning outcomes [55,56,57], it is clear that many variables contribute to the likelihood of success.

The aims of this research were to investigate the knowledge and attitudes of abattoir stakeholders in China, to assess whether training influenced knowledge and attitudes, and to determine whether delivery mode of training to the facilitators affected learning outcomes for the trainees.

## 2. Materials and Methods

### 2.1. Training Workshops

Under the auspices of the AWSP, a training package on improving animal welfare during slaughter was designed and provided to senior livestock stakeholders to facilitate local workplace stakeholder training workshops within the abattoir in which they worked. Top-down training was undertaken to improve abattoir stakeholder knowledge of, and attitudes towards, livestock transport and slaughter. The intention was to train abattoir staff (“facilitators”) working at managerial levels of team leader or higher, who could implement improvements in their own workplaces by training other staff (“trainees”). Facilitator training was undertaken using one of two delivery modes; a workshop held in March 2018, and remote training via posting of the materials with instructions. Abattoirs and abattoir staff can be difficult to access due to biosecurity and safety concerns, therefore, potential facilitators were identified through government or academic colleagues who had existing connections with abattoirs.

We invited 16 potential trainers to attend the “facilitator” workshop. Training and attendance costs (e.g., travel) were provided to the participants, but on completion of the workshop each attendee was considered a “facilitator” and expected to run their own training using the same materials for at least 20 attendees in their own workplace. In the end, through the initial invitees extending the invitation to others, about 30 attendees came to the “facilitator” workshop, from four separate abattoirs. Two of these were pig abattoirs in Luoyang and Yichuan (both Henan province) and the other two were chicken abattoirs in Kaifeng (Henan Province) and Huizhou (Guangdong Province). The workshop was based on the World Organisation for Animal Health (OIE) Terrestrial Animal Health Code, Section 7: Animal Welfare (OIE World Organisation for Animal Health, 2015) and included information on animal welfare issues for livestock in abattoirs, methods to address these, international accreditation schemes, and OIE guidelines. The workshop also discussed guidelines for running stakeholder workshops, the importance of stunning prior to slaughter, and local industry examples of successful implementation of stunning standards. The facilitators’ workshop was presented in English or Mandarin by a selection of international and local Chinese animal welfare experts and AWSP team members, with each presentation being translated by a native Mandarin speaker from English to Mandarin where necessary. Following the facilitator workshop series, the four facilitator groups trained 95 other employee stakeholders under their direct supervision (“trainees”) using the project workshop materials they were provided on a USB, and via an online link. Facilitators were given guidance on how to present the training materials, and generally recruited staff for their workshop by an announcement within the abattoir. The facilitators ran their own workshops approximately three months after their training. Another, separate batch of training was completed using remote delivery of training materials. A project collaborator at the Southern China Agricultural University distributed the training resources to nine facilitators from different abattoirs who did not attend the workshops but agreed to complete the training resources in their own time and present the workshop to employees in their workplace. These abattoirs were from different parts of Guangdong province (Huizhou, Maoming, Yunfu, and two each in Guangzhou, Shenzhen, and Jiangmen). Effectiveness of the top-down training was determined using questionnaires distributed by facilitators to their trainees.

### 2.2. Questionnaire

The questionnaire was adapted from research developed, validated, and published under the AWSP [18,54,58]. The original questionnaire, and the adapted one used in this study, were developed in collaboration with researchers from China. The questionnaire was written in English before being translated into Zhōngwén (written Chinese) by a professional translator. To verify the accuracy of the translation, it was back-translated by an academic in the Chinese Southern Agricultural University who had not viewed the English version. The original and back-translated English versions were compared for accuracy and retention of meaning and were found to be acceptably similar.

The questionnaire was comprised of three sections. Part A included demographic questions such as gender, age, level of education, length of involvement with the industry, and what type of area they had lived in for most of their life (rural/town/suburban/city). Respondents were asked to provide a broad rating of animal welfare standards at their workplace (5-point scale from Very Good to Very Bad), and to rate their own knowledge of livestock slaughter (5-point scale from Very high to Very low). This section also asked how they gain their professional knowledge (Formal qualifications/Workplace employment/Personal interest/Friends and acquaintances).

Part B of the questionnaire assessed their knowledge of slaughter and transport practices. Questions were in a multiple-choice format and based on content provided within training workshops. This section was scored for accuracy with each correct answer awarded one mark, an incorrect answer awarded zero marks and a total provided out of 15 marks to represent a Knowledge Score. A higher Knowledge Score indicated more accurate knowledge.

Part C assessed the attitudes of respondents to fifteen statements about animal welfare, slaughter and transport. Responses were recorded on a 5-point Likert scale of “Strongly disagree”, “Disagree”, “Neither agree nor disagree”, “Agree”, and “Strongly agree”.

Stakeholders were all over 18 years of age and were informed that their participation was voluntary, and their answers would be used for research purposes. In total, questionnaires from 308 individuals were collected. A total of 161 were completed prior to training (58 from trainees whose facilitators attend on-site training (“Direct” training group), and 103 from trainees whose facilitators trained using self-directed materials (“Remote” training group). A total of 147 were completed after training (49 “Direct” respondents and 98 “Remote” respondents). Questionnaires were completed in paper format and were collected by the primary researcher or by the facilitators who then mailed them to the primary researcher, X.L.

### 2.3. Ethics

This study was approved by the University of Queensland Human Ethics Committee (approval number 2017001612).

### 2.4. Statistical Analysis

Microsoft Excel was used for checking and cleaning of data (Microsoft ^®^ Excel ^®^, Version 16.0.4849.1000, Microsoft Corporation, Washington, USA) and R was used for analysis [59] with packages psych [60], plyr [61], factoextra [62], car [63], agricolae [64], and MASS [65].

Descriptive statistics were generated using the describe and describeBy functions, with tapply used for calculating variances. Differences between pre- and post-training groups for self-rating questions (Q7 & Q8) and attitudinal questions (Q26–Q40) were determined using a Kruskal–Wallis test with the Kruskal.test() function. Knowledge differences between pre- and post-training groups were assessed using a two-way ANOVA that also included TrainingSession (“Direct” or “Remote”) as an explanatory variable. Assumptions of the ANOVA were checked using residual-fitted, scale-location, residual-leverage, and QQ-plots with the plot function. Relationships between attitudinal questions were explored using a Principal Component Analysis with the prcomp() function, and visualization with the fviz_eig and fviz_pca_var functions. Principal Component Analysis is a multi-variate analysis that can be used to reduce the dimensions of a dataset by finding new variables (“components”) which are combinations of existing variables based on the amount of variation [66].

Multi-variable linear models were used to assess the influence of explanatory, demographic variables (Gender, Age, Education, Length of Involvement in the Industry, Type of Living Area) on attitude PC1 and PC2 and the Knowledge Score, using the lm function. When including Gender, the third gender option of non-disclosed was removed, as all respondents except one identified as male or female, and therefore the third gender was not able to be included meaningfully. To extract summary details, the Anova function was used and minimum adequate models were determined using stepwise selection with the stepAIC function.

## 3. Results

### 3.1. Demographic Information of Respondents

In total, 308 stakeholders completed the training and survey, 161 stakeholders before training and 147 after. Of the respondents, almost 70% were male, and almost 80% were aged between 26 and 45 years (Table 1). Approximately equal numbers worked with pigs and chickens, and over half of all respondents had worked in the industry for less than one year (Table 1). Most respondents (56.8%) lived in rural regions, and a third had only completed middle school as their highest education level (Table 1).

### 3.2. Self-Rating Questions

On a mean (±se) score between 1 (Very good) and 5 (Very poor), respondents rated animal welfare within their workplace as 2.6 (±0.05) and their own knowledge about animal slaughter as 3.1 (±0.05). Self-rating of slaughter knowledge was better in the post-training group compared to the pre-training group (*p* = 0.01), while rating of welfare within the workplace did not differ (*p* = 0.07) (Table 2).

### 3.3. Knowledge Scores

**Figure 1:**
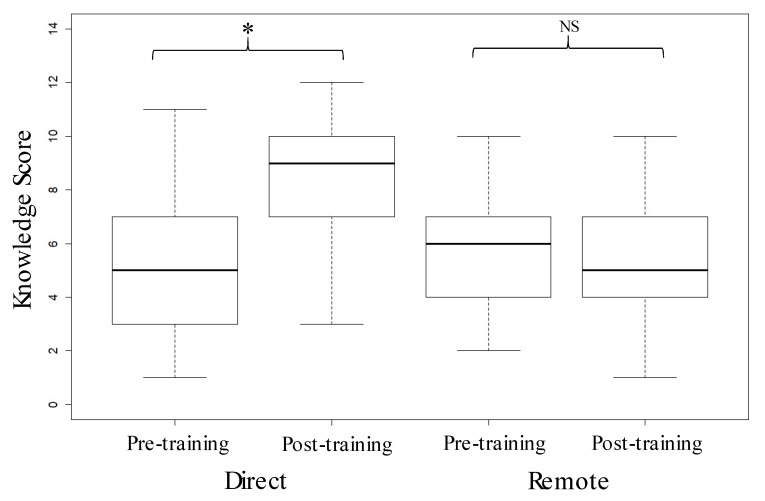
The effect of training delivery method (Direct or Remote) before (Pre-) and after (Post-) training on Knowledge Scores. Significant (* = *p* < 0.05) and non-significant (NS = *p* > 0.05) changes between before and after scores are indicated above the brackets.

**Figure 2:**
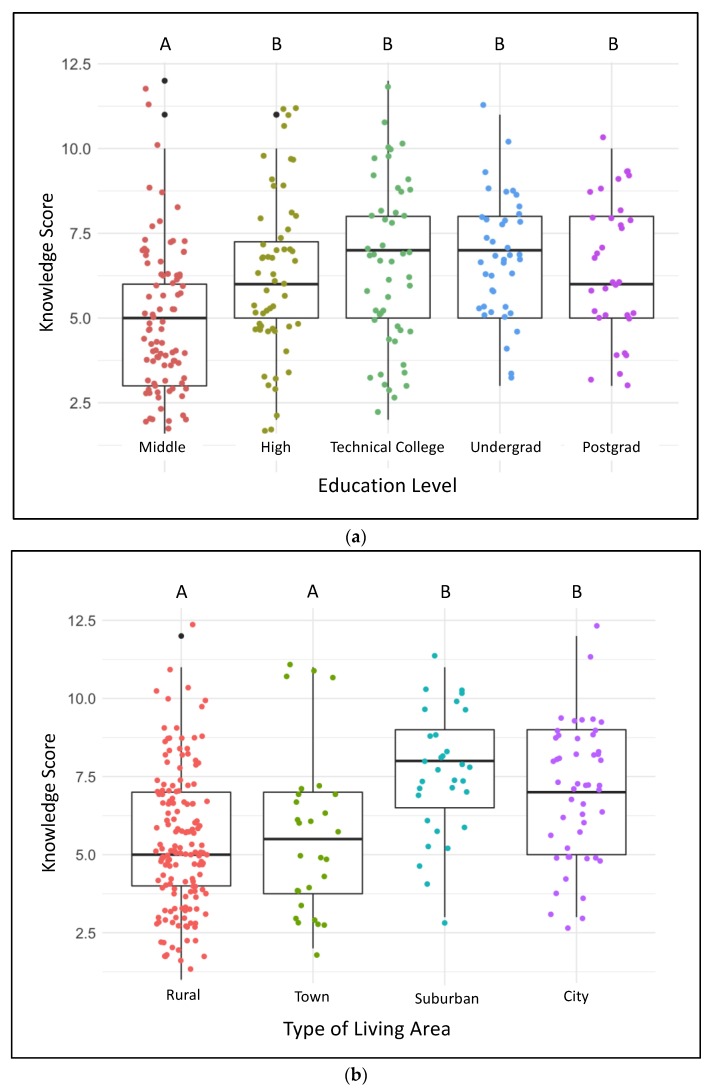
Boxplots of Knowledge Score for respondents with different education levels (**a**) and living areas (**b**). Uppercase letters indicate differences between groups (groups with different letters are significantly different at *p* < 0.05 from each other). Individual datapoints within the group are depicted.

Knowledge Scores had a possible range of 0 (none correct) to 15 (all correct). The mean (±se) score was 6.0 (±2.4) and individual scores ranged from 1 to 12. Knowledge Scores were affected by training, with scores in the Post-training group being higher than those from the Pre-training group (*p* < 0.006) (Table 2). There was however a difference in Knowledge Scores according to Training Type (F_1,306_ = 20.1, *p* < 0.0001) and an interaction between Training Type and whether the respondent was Pre- or Post-training (F_1,304_ = 51.63, *p* < 0.0001) (Figure 1). Direct training (trainees whose facilitator was trained in a classroom setting) resulted in higher post-training scores, when compared with pre-training scores. Remote training (a training package was mailed to the facilitator who then carried out training) resulted in no pre- to post-training changes in scores (Figure 1). A multi-variable analysis of Knowledge Score with demographic variables found an effect of Education (*p* = 0.005) and Living Area (*p* = 0.005) (Table 3). Respondents with only a middle school education had a lower Knowledge Score than other respondents (Figure 2a), and those living in Suburban and City areas had a higher score than those living in Rural areas or Towns (Figure 2b).

### 3.4. Attitudinal Influences

The attitudinal section aimed to test attitudes towards a range of contexts that were applicable to animal welfare during slaughter. Questions 26 and 27 asked if the welfare of animals during slaughter and transportation, respectively, were important to the respondents. A third of respondents (33%) indicated that welfare during slaughter was neither important nor unimportant, and a similar rating (36%) was given for welfare during transportation (Table 4), while 39% and 41% of respondents agreed that welfare was important during slaughter and transport, respectively. Less than 15% disagreed or strongly disagreed that animal welfare was important to them in these contexts (Table 4). Close to half of all respondents indicated that, in their own workplace, animal welfare was satisfactory during slaughter or transportation, while around 41% neither agreed nor disagreed with the statement (Table 4). Over half of the respondents (58.8%) were confident that they could make improvements to animal welfare, while a similar proportion had tried to make improvements in the past (55.2%) or intended to do so in the future (58.4%) (Table 4).

A large proportion of participants disagreed or strongly disagreed with the killing of dependent animals (73.1%), allowing animals to experience pain during slaughter (71.4%), allowing animals to see each other being slaughtered (74.1%), and transportation without sufficient space and facilities (67.5%). Around half of respondents agreed/strongly agreed that food and water should be provided before and during transport (52.9%) and disagreed/strongly disagreed that animals should be killed when seriously injured or ill (55.2%). Less than 10% of respondents agreed with the use of products obtained from animals who had died naturally (Table 4).

Most attitudinal responses were not different between the pre-training and post-training groups however two items were significantly different. The post-training group had more confidence that they could make changes to animal welfare (*p* = 0.03) and had a higher score indicating past attempts to improve animal welfare (*p* = 0.006) (Table 2).

**Figure 3:**
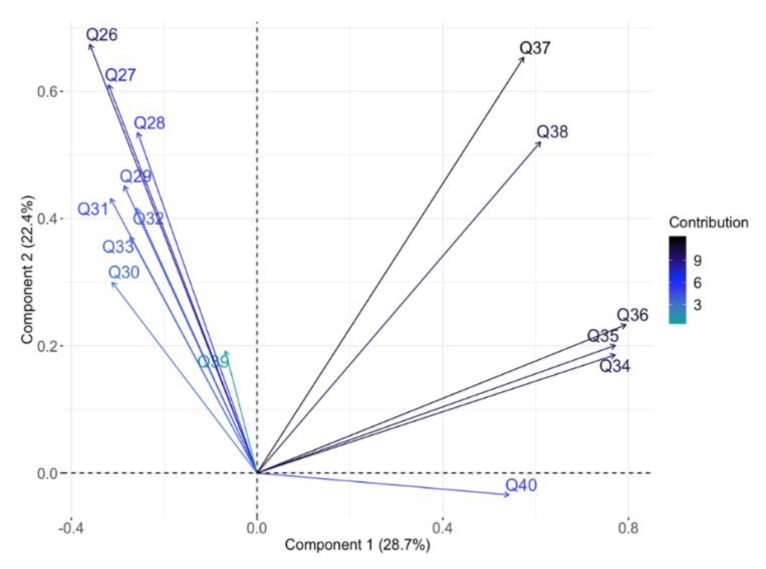
Loading plot for components 1 (28.7% of variance) and 2 (22.4% of variance) of attitudinal questions, showing the dichotomization of responses along Principal Component 1 into two groups: (i) Questions 26–33 and 39 and (ii) Questions 34–38 and 40.

**Figure 4:**
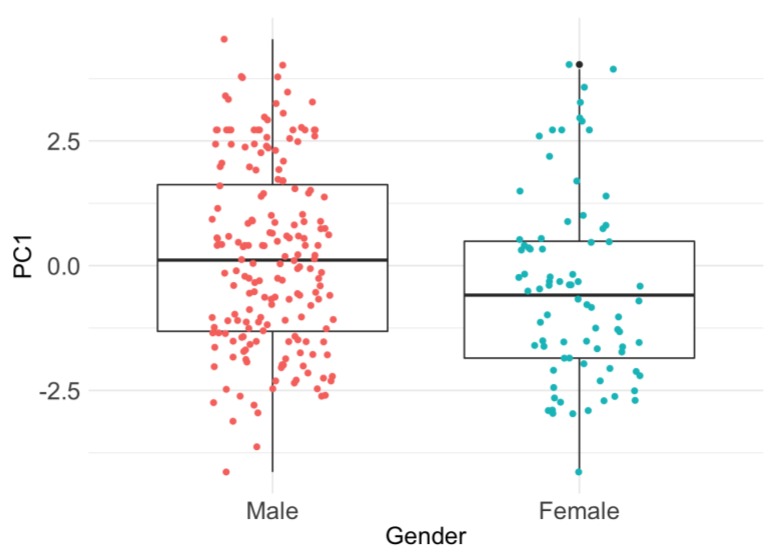
Boxplots of Principal Component 1 (PC1) loadings of attitudinal items for male and female respondents. Interpretation of the contributing PCA variables indicated that PC1 and attitudes towards animal welfare were negatively associated (more negative PC1 loadings for individuals reflecting more positive attitudes towards animal welfare).

A principal components analysis of the attitudinal responses suggested two main components (PC1 and PC2), explaining 28.7% and 22.4% of the variance, respectively. PC1 split clearly into two main groups. The first group comprised Q26 to Q33 and Q39, and the second, Q34 to Q38 and Q40 (Figure 3). Examination of these groupings suggests that PC1 indicates the level of support for animal welfare. Group one (Q26–33, 39) includes statements such as “The welfare of animals during slaughter is important to me”, “I intend to make improvements to the welfare of the animals in my care”, and “The provision of food and water to animals before or during transport” (Figure 3, Table 4). A high score for these items (4 or 5) indicates agreement with the statement, therefore, participants who scored strongly towards group one (a negative score on PC1) tended towards positive attitudes for animal welfare. The second group (Q34–38, 40) included statements such as “Allowing animals to experience pain during slaughter”, “Letting animals see each other being slaughtered”, and “Transporting animals with insufficient space and improper facilities”. Again, a high score indicates agreement with the statements so respondents who scored highly in group two (a positive score on PC1) tended towards negative attitudes with regards to animal welfare. PC1 was not affected by whether the respondents were pre- or post-training (F_1,268_ = 1.60, *p* = 0.21), nor by Knowledge Score (F_1,268_ = 1.01, *p* = 0.31). A multi-variable analysis using PC1 as the outcome variable and demographic information (sex, age, education, length of involvement in the industry, and type of living environment) as the explanatory variables found that only gender was a significant predictor of attitude (Table 3; Figure 4). Female respondents had a more negative PC1 loading compared to males (*p* = 0.009, Table 3), suggesting a more positive attitude towards animal welfare (Figure 4).

**Figure 5:**
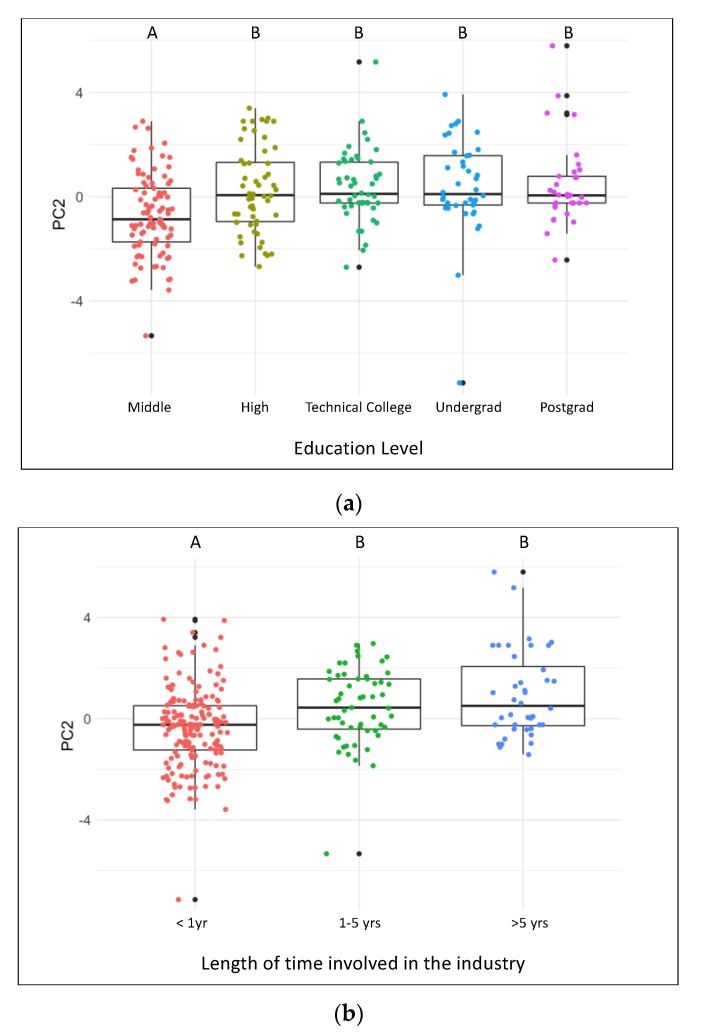
Boxplots of PC2 loadings of attitudinal items for respondents with different education levels (**a**) and length of time involved in the industry (**b**). Uppercase letters indicate differences between groups (groups with different letters are significantly different *p* < 0.05 from each other).

Interpretation of PC2 was less evident, despite the amount of variance explained by this component. Loadings of items onto PC2 were continuous rather than discrete, so the items did not separate into easily explained groupings (Figure 3, Table 4). PC2 was not affected by whether respondents were pre- or post-training (H = 0.08, df = 1, *p* = 0.78), but was affected by Knowledge Score (β = 0.26, *p* < 0.0001). A multi-variable analysis using PC2 as the outcome variable and demographic information as the explanatory variables found a significant relationship with Education and Length of involvement in the industry (Table 3). Respondents with an education level higher than middle school (Figure 5a) and industry involvement greater than one year had a more positive PC2 loading (Figure 5b).

## 4. Discussion

The results from this study highlight important aspects regarding the demographics of abattoir stakeholders in China, their attitudes towards animals in the abattoir context, and the effect of training on their knowledge. More than half of the participants in this study had less than a year’s experience in the industry and a third had not completed high school. This suggests that the staff turnover in this industry is quite high, and employment of this kind is not particularly desirable. Abattoir work is often considered to be “dirty work” [67], work that is viewed with social disapproval or unease [68,69], and employment in such jobs can be stigmatized [67]. The perception of dirty work is not uniform and has cultural influences. Zookeeping, for example, can be viewed as either glamorous or dirty [70], and while most studies on “dirty work” have been conducted in Western cultures, the concept is also evident in other countries, including China [71,72]. This may have implications for the effectiveness of training initiatives, and for the improvement of animal welfare in these industries. High staff turnovers require job-specific training to be frequent and efficient, and where there is a high proportion of staff members with lower levels of education, training must be presented in an appropriately accessible way. Education did affect attitude scores in this study, with less educated respondents having increased scores in PC2 and therefore a less benign attitude to animal welfare; a similar result has been found in previous abattoir research regarding disease prevention [73]. Importantly, education levels may affect adherence of staff to workplace protocols, as has been found for compliance with abattoir food safety practice [74].

Three-quarters of the participants indicated that they gained their professional knowledge from their workplace. This finding aligns with meat processing industries outside China. One Australian study [75] found that most job-specific training was conducted by experienced workers within the same facility, and a 2010 government report [76] suggested this style of training had been implemented due to high staff turnover rates. This creates both challenges and opportunities in terms of animal welfare-specific training. The practical implications are that training initiatives will need to be widely dispersed in order to reach a mass of abattoir workers and regularly conducted to account for the high turnover of staff. However, if workplace trainers are upskilled in animal welfare content, then welfare-friendly protocols could be implemented alongside other workplace specific training such as food safety and occupational risk management.

The results of our study indicated that there was some beneficial effect of training on knowledge as respondents in the post-training group had a higher knowledge score than those in the pre-training group, which aligns with previous research using similar methodologies [77]. The post-training respondents also gave a higher self-rating of their knowledge and indicated they were more confident in their ability to create change. Although these differences were statistically significant, the absolute change in scores was not large, suggesting there is room for improvement in training. Importantly, the method of delivery had a large impact on the difference between pre- and post-training scores. There was no difference between the pre- and post-training scores for the remotely-trained group, but there was for the group whose facilitators were originally trained in the classroom setting. Remote training can be a cost-effective way to deliver educational initiatives [78] and can be more accessible for people based in remote locations than classroom-based training [79] but learning success can be varied [80]. Many educational institutions now offer distance education opportunities, but the training initiative in our study differ from these in two key ways. Only the facilitator training was delivered remotely or in person, although this clearly had a flow-on effect to the trainees. Additionally, most modern distance training programs incorporate online tools and other technologies to assist with engagement and learning [81,82,83]. This may be an avenue for further development of welfare training in the meat industry.

Training had no effect on attitudes towards animal welfare but did appear to improve participant confidence and their assessment of past efforts, which may indicate that they better recognize when past actions were relevant to welfare. In contrast to training, attitudes were most influenced by gender, which aligns with similar previous research [77]. There were, however, some important attitudinal aspects that emerged from this survey. Around 10% of respondents stated that welfare during slaughter and transport was not important, and more than one third indicated that it was neither important nor unimportant. Despite this, a strong welfare stance emerged on a few specific issues. Most respondents disagreed with the killing of dependent young, pain during slaughter, and on allowing animals to see other animals being killed. Also, most did not agree with using animals that had died naturally, which may be influenced by aspects of food safety. Food safety is a recognized concern for Chinese citizens [52] and poor animal welfare can increase the risk of food contamination or disease susceptibility [84]. These aligning issues may create valuable opportunities for the improvement of animal welfare [85].

The attitudinal items that comprised component two (PC2) of the Principal Component Analysis were influenced by participant knowledge and experience. PC2 was positively related to Knowledge Score, since those with more education or experience in the industry also had higher PC2 scores. This suggests that this component was either a result of their knowledge, education, and/or experience, or alternatively it may reflect an underlying latent trait that influences these, such as personality, confidence, early life history, or their family economic status [86]. For example, an Australian government report on meat processing identified two main groups of workers within that industry [76]. One group tended to be transient workers, and the second group were more permanent staff with intentions to continue in the industry. It may be that a similar underlying difference is also present in the current study. Previous research indicates that that the longer a stakeholder is involved in the industry, the more important they perceive aspects of slaughter and transport to be, such as stunning, and maintaining loss of consciousness after stunning; similar results were found regarding stunning for those with higher education levels [87]. Therefore, it may be valuable for future studies to consider retention of staff in this industry as an indirect method of also improving animal welfare.

Limitations of this study are that the same participants were not surveyed before and after, and there is a lack of information on how facilitators conducted their training sessions within their workplace.

Furthermore, China is a large and diverse country with numerous regional differences, including economic, work culture, wage inequality, and skill premium (the salary gap between skilled and unskilled labor) [88,89,90]. Useful future research would include a detailed study of training approaches or effects by region.

Future directions could investigate how welfare training could be merged with existing workplace training, and how current distance educational tools could be employed to improve learning outcomes. Known existing priorities such as food safety or quality could be taken advantage of to create alignment with animal welfare training. Future studies should also link training and knowledge improvement with behavioral change by abattoir stakeholders and improved animal welfare outcomes.

## Figures and Tables

**Table 1:** Demographic information of respondents and assessment of animal welfare of all stakeholders completing the survey (*n* = 308). Where percentages sum to less than 100, some answers were blank or not valid. Where percentages sum to more than 100, respondents could choose more than one answer.

Gender	N (%)	Age	N (%)
Male	214 (69.5)	18–25 years	48 (15.6)
Female	93 (30.2)	26–35 years	119 (38.6)
Other	1 (0.3)	36–45 years	111 (36)
		46–55 years	24 (7.8)
**Species working with**		>55 years	6 (2.0)
Pigs	157 (51.0)		
Chickens	151 (49.0)	**Type of living area**	
		Rural	175 (56.8)
**Level of education**		Town	32 (10.4)
Middle school	105 (34.1)	Suburb	38 (12.3)
High school	65 (21.1)	City	62 (20.1)
Technical/trades college	59 (19.2)		
Undergraduate degree	42 (13.6)	**Length of involvement in industry**	
Postgraduate degree	33 (10.7)	<1 year	179 (58.1)
Other	2 (0.65)	1–5 years	69 (22.4)
		>5 years	59 (19.2)
**Role in industry**			
Transportation	122 (39.6)	**Method of gaining knowledge**	
Stunning	17 (5.5)	Formal qualifications	93 (30.2)
Unloading	28 (9.1)	Workplace	234 (76.0)
Lairage	39 (12.7)	Personal interest	80 (26.0)
Carcass processing	45 (14.6)	Friends/acquaintances	55 (17.9)
Business manager	81 (26.3)		

**Table 2:** Differences in knowledge scores, self-rating, and mean attitude scores, before and after training.

	**Mean Score (Before)**	**Mean Score (After)**	**Pooled SE**	**F Statistic (df = 1,306)**	***p* Value**
**Knowledge score (0 = none correct to 15 = all correct)**	5.63	6.37	0.65	7.71	0.006
**Self-rating questions (1 = Very good to 5 = Very poor)**	**Mean Score (before)**	**Mean Score (after)**	**Pooled SE**	**H statistic (df = 1)**	***p* value**
Self-rating of workplace’s animal welfare	2.70	2.51	0.11	3.26	0.07
Self-rating of level of slaughter knowledge	3.23	2.91	0.09	6.53	0.01
**Attitudinal questions (1 (Strongly disagree) to 5 (Strongly agree))**					
The welfare of animals during slaughter is important to me	3.48	3.55	0.11	0.86	0.35
The welfare of animals during transport is important to me	3.5	3.64	0.10	2.52	0.11
The welfare of animals during slaughter is satisfactory in my workplace	3.49	3.5	0.08	0.10	0.75
The welfare of animals while being transported is satisfactory in my workplace	3.43	3.56	0.07	2.37	0.12
Most people who are important to me would approve of me making improvements to the welfare of animals in my care	3.47	3.56	0.07	0.52	0.47
I intend to make improvements to the welfare of the animals in my care	3.6	3.71	0.06	2.32	0.13
I am confident that I can make improvements to the welfare of animals	3.57	3.73	0.06	4.81	0.03
In the past I have tried to make improvements to the welfare of the animals in my care	3.42	3.68	0.07	7.51	0.006
Killing animals that are still dependent on their parent	1.91	1.94	0.10	0.073	0.79
Allowing animals to experience pain during slaughter	2.06	1.9	0.10	2.12	0.15
Letting animals see each other being slaughtered	1.98	1.81	0.10	3.42	0.06
Killing animals when they are seriously injured or ill	2.36	2.31	0.16	0.42	0.52
Using animals that have died naturally for consumption	1.99	1.97	0.14	0.55	0.46
The provision of food and water to animals before or during transport	3.31	3.45	0.11	2.77	0.10
Transporting animals with insufficient space and improper facilities	2.2	2.23	0.10	0.01	0.92

**Table 3:** Minimum Adequate Model (MAM) summaries of principal components one and two obtained from a PCA of attitudinal responses, and Knowledge score using backward stepwise selection. Change (Δ) in AIC is the decrease in Akaike Information Criterion from the full model (~Gender + Age + Education + Time in industry, Type of living area).

Outcome Variable	MAM	Δ AIC	Test Statistic	*p*-Value
PC1	~Gender	2.84	F_(1,266)_ = 6.79	*p* = 0.009
PC2	~Education	5.92	F_(4,261)_ = 6.00	*p* < 0.001
	+Time in Industry		F_(2,261)_ = 12.21	*p* < 0.0001
Knowledge Score	~Education	3.17	F_(4,258)_ = 3.78	*p* = 0.005
	+Time in Industry		Not sig.	
	+Living Area		F_(3,258)_ = 4.35	*p* = 0.005

**Table 4:** Responses to attitudinal questions. Loadings on Principal Components 1 and 2, Number of valid responses, and percentage of responses.

Question/Statement	PC1 Loading	PC2 Loading	Number of Responses	Strongly Disagree (%)	Disagree (%)	Neither Disagree nor Agree (%)	Agree (%)	Strongly Agree (%)
The welfare of animals during slaughter is important to me	−0.19	0.41	304	3.2	9.4	33.4	38.6	14.0
The welfare of animals during transport is important to me	−0.17	0.37	303	1.9	6.8	35.7	41.2	12.7
The welfare of animals while being slaughtered is satisfactory in my workplace	−0.14	0.32	302	2.6	4.9	41.9	39.0	9.7
The welfare of animals while being transported is satisfactory in my workplace	−0.15	0.27	298	1.3	6.5	41.2	39.0	8.8
Most people who are important to me would approve of me making improvements to the welfare of animals in my care	−0.17	0.18	301	1.3	4.9	41.2	42.9	7.5
I intend to make improvements to the welfare of animals in my care	−0.17	0.26	301	0.6	2.9	35.7	48.7	9.7
I am confident that I can make improvements to the welfare of animals in my care	−0.14	0.24	300	0.6	3.9	34.1	49.7	9.1
In the past I have tried to make improvements to the welfare of animals in my care	−0.15	0.22	298	1.9	5.2	34.4	48.7	6.5
Killing animals that are still dependent on their parents	0.41	0.11	302	40.3	32.8	17.5	7.5	0
Allowing animals to experience pain during slaughter	0.41	0.12	303	35.4	36.0	19.8	7.1	0
Letting animals see each other getting slaughtered	0.42	0.14	303	40.3	33.8	18.5	5.5	0.3
Killing animals when they are seriously injured or ill	0.31	0.39	302	30.8	24.4	23.4	17.9	1.6
Using animals that have died naturally for products	0.32	0.31	302	43.2	25.0	21.8	4.9	3.2
The provision of food and water to animals before and during transport	−0.04	0.12	300	4.2	15.3	25.0	45.1	7.8
Transporting animals with insufficient space and improper facilities	0.29	−0.02	304	21.4	46.1	20.8	9.4	1.0
